# Holmium dodeca­iodidoiron-*octa­hedro*-hexa­holmium, {FeHo_6_}I_12_Ho

**DOI:** 10.1107/S1600536809001640

**Published:** 2009-01-23

**Authors:** Kathrin Daub, Gerd Meyer

**Affiliations:** a Institut für Anorganische Chemie, Universität zu Köln, Greinstrasse 6, D-50939 Köln, Germany

## Abstract

Single crystals of {FeHo_6_}I_12_Ho were obtained during the reaction of HoI_3_ with metallic holmium and iron in a sealed tantalum container. The crystal structure consists of isolated holmium clusters encapsulating a single Fe atom, {FeHo_6_} (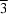 symmetry). The rare earth metal atoms are surrounded by 12 edge-capping and six terminal iodide ligands that either connect the clusters to each other directly or *via* HoI_6_ octa­hedra (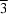 symmetry).

## Related literature

Reduced rare earth metal halides without and with metal clusters have been reviewed several times, see, for example: Corbett (1973 ▶, 1996 ▶, 2000 ▶, 2006 ▶); Hughbanks & Corbett (1988 ▶); Meyer (1988 ▶, 2007 ▶); Meyer & Wickleder (2000 ▶); Simon (1981 ▶); Simon *et al.* (1991 ▶); Wiglusz *et al.* (2007 ▶). For the synthesis of the starting material HoI_3_, see: Meyer (1991 ▶). Isotypic structures have been reported by Hohnstedt (1993 ▶), {CHo_6_}I_12_Ho, and Palasyuk *et al.* (2006 ▶), {FePr_6_}I_12_Pr.

## Experimental

### 

#### Crystal data

FeHo_7_I_12_
*M* *_r_* = 2733.16
Trigonal, 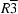

*a* = 15.2973 (17) Å
*c* = 10.6252 (16) Å
*V* = 2153.3 (5) Å^3^
*Z* = 3
Mo *K*α radiation
μ = 32.43 mm^−1^
*T* = 293 (2) K
0.2 × 0.2 × 0.2 mm

#### Data collection

Stoe IPDS-II diffractometer
Absorption correction: numerical [*X-RED* (Stoe & Cie, 2001 ▶) and *X-SHAPE* (Stoe & Cie, 1999 ▶)] *T*_min_ = 0.027, *T*_max_ = 0.071
6920 measured reflections
1166 independent reflections
861 reflections with *I* > 2σ(*I*)
*R*_int_ = 0.115

#### Refinement

*R*[*F* ^2^ > 2σ(*F* ^2^)] = 0.039
*wR*(*F* ^2^) = 0.096
*S* = 0.97
1166 reflections
32 parameters
Δρ_max_ = 2.36 e Å^−3^
Δρ_min_ = −2.44 e Å^−3^

### 

Data collection: *X-AREA* (Stoe & Cie, 2001 ▶); cell refinement: *X-AREA*; data reduction: *X-AREA*; program(s) used to solve structure: *SHELXS97* (Sheldrick, 2008 ▶); program(s) used to refine structure: *SHELXL97* (Sheldrick, 2008 ▶); molecular graphics: *DIAMOND* (Brandenburg, 2005 ▶); software used to prepare material for publication: *SHELXL97*.

## Supplementary Material

Crystal structure: contains datablocks I, global. DOI: 10.1107/S1600536809001640/wm2215sup1.cif

Structure factors: contains datablocks I. DOI: 10.1107/S1600536809001640/wm2215Isup2.hkl

Additional supplementary materials:  crystallographic information; 3D view; checkCIF report

## Figures and Tables

**Fig. 1.**
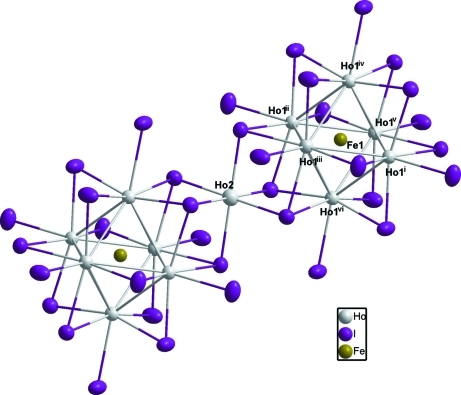
: {FeHo6} clusters connected via HoI6 units, drawn with displacement ellipsoids at the 90% probability level [Symmetry codes: (i) 1 + y, 1 - x + y, 1 - z; (ii) 1 - y, -1 + x-y, z; (iii) -x + 8/3, -y + 1/3, -z + 4/3; (iv) x-y, -1 + x, 1 - z; v) 2 - x, -y, 1 - z; vi) 2 - x + y, 1 - x, z.]

## supplementary crystallographic information

### Comment

Rare earth cluster compounds of the general formula {*Z(RE)*_6_}I_12_*RE*, where *Z* is an interstitial transition metal or main group element and *RE* is a rare earth element, have been well explored by Hughbanks and Corbett (1988) for *RE* = Sc, Y, Pr and Gd. Additionally, compounds of the formula {*Z(RE)*_6_}I_12+_*_y_A_x_*, where *A* is an alkali metal (Rb or Cs) with *x* = 1–4 and *y* = 0–1 and *Z* = C, C_2_, are known for the rare earth elements Pr and Er that were compiled and studied by Meyer & Wickleder (2000) and Wiglusz *et al.* (2007). With {FeHo_6_}I_12_Ho we were able to extend the knowledge of this structure type to the element holmium, where only {CHo_6_}I_12_Ho had been synthesized previously by Hohnstedt (1993). Other reviews of reduced rare earth metal halides without and with metal clusters were given, for example, by Corbett (1973, 1996, 2000, 2006), Meyer (1988, 2007), Meyer & Wickleder (2000), Simon (1981) and Simon *et al.* (1991).

The structure of {FeHo_6_}I_12_Ho is isotypic with {FePr_6_}I_12_Pr (Palasyuk *et al.*, 2006) and consists of isolated {FeHo_6_} clusters, *i.e.* the metal atoms are not shared with other clusters. The {FeHo_6_} cluster core is surrounded by twelve edge-capping and six terminal iodide ligands that either connect the clusters to each other directly or *via* HoI_6_ octahedra (Fig. 1). In {FeHo_6_}I_12_Ho, the {FeHo_6_} octahedra have 3 symmetry, only slightly deviating from ideal octahedral symmetry. The Ho—Ho distances range from 3.6394 (11) to 3.7297 (12) Å. The Ho—I distances vary between 3.0106 (9) and 3.3116 (12) Å.

### Experimental

Black, almost cubic crystals of {FeHo_6_}I_12_Ho were obtained by the reaction of HoI_3_ (200 mg) with holmium powder (84 mg, Chempur, 99.9%) and iron powder (10 mg, Merck, p.a.) in a tantalum container at 1273 K for 200 h. HoI_3_ had been synthesized from stoichiometric amounts of holmium and iodine, followed by sublimation in high vacuum for purification (Meyer, 1991). Due to air and moisture sensitivity of both reagents and products, all handlings were carried out in an argon-filled glove box (M. Braun, Garching, Germany).

### Refinement

The displacement parameter for the Fe atom was refined isotropically. The highest peak (2.36 e Å^-3^) in the final difference Fourier map is 1.20 Å from atom Ho1 and the deepest hole (-2.44 e Å^-3^) is 2.40 Å from the same atom.

### Crystal data

**Table:**

FeHo_7_I_12_	*D*_x_ = 6.323 Mg m^−^^3^
*M**_r_* = 2733.16	Mo *K*α radiation, λ = 0.71073 Å
Trigonal, *R*3	Cell parameters from 1775 reflections
Hall symbol: -R 3	θ = 1.9–28.2°
*a* = 15.2973 (17) Å	µ = 32.43 mm^−^^1^
*c* = 10.6252 (16) Å	*T* = 293 K
*V* = 2153.3 (5) Å^3^	Cubic, black
*Z* = 3	0.2 × 0.2 × 0.2 mm
*F*(000) = 3393	

### Data collection

**Table:**

Stoe IPDS-II diffractometer	1166 independent reflections
Radiation source: fine-focus sealed tube	861 reflections with *I* > 2σ(*I*)
graphite	*R*_int_ = 0.115
ω scans	θ_max_ = 28.1°, θ_min_ = 2.5°
Absorption correction: numerical [*X-RED* (Stoe & Cie, 2001) and *X-SHAPE* (Stoe & Cie, 1999)]	*h* = −20→19
*T*_min_ = 0.027, *T*_max_ = 0.071	*k* = −19→20
6920 measured reflections	*l* = −14→14

### Refinement

**Table:**

Refinement on *F*^2^	Primary atom site location: structure-invariant direct methods
Least-squares matrix: full	Secondary atom site location: difference Fourier map
*R*[*F*^2^ > 2σ(*F*^2^)] = 0.039	*w* = 1/[σ^2^(*F*_o_^2^) + (0.047*P*)^2^] where *P* = (*F*_o_^2^ + 2*F*_c_^2^)/3
*wR*(*F*^2^) = 0.096	(Δ/σ)_max_ = 0.001
*S* = 0.97	Δρ_max_ = 2.36 e Å^−^^3^
1166 reflections	Δρ_min_ = −2.44 e Å^−^^3^
32 parameters	Extinction correction: *SHELXL97* (Sheldrick, 2008), Fc^*^=kFc[1+0.001xFc^2^λ^3^/sin(2θ)]^-1/4^
0 restraints	Extinction coefficient: 0.00035 (3)

### Special details

**Table:**

Geometry. All e.s.d.'s (except the e.s.d. in the dihedral angle between two l.s. planes) are estimated using the full covariance matrix. The cell e.s.d.'s are taken into account individually in the estimation of e.s.d.'s in distances, angles and torsion angles; correlations between e.s.d.'s in cell parameters are only used when they are defined by crystal symmetry. An approximate (isotropic) treatment of cell e.s.d.'s is used for estimating e.s.d.'s involving l.s. planes.
Refinement. Refinement of *F*^2^ against ALL reflections. The weighted *R*-factor *wR* and goodness of fit *S* are based on *F*^2^, conventional *R*-factors *R* are based on *F*, with *F* set to zero for negative *F*^2^. The threshold expression of *F*^2^ > σ(*F*^2^) is used only for calculating *R*-factors(gt) *etc*. and is not relevant to the choice of reflections for refinement. *R*-factors based on *F*^2^ are statistically about twice as large as those based on *F*, and *R*- factors based on ALL data will be even larger.

### Fractional atomic coordinates and isotropic or equivalent isotropic displacement parameters (Å^2^)

**Table:**

	*x*	*y*	*z*	*U*_iso_*/*U*_eq_	
Ho1	1.15739 (5)	0.04355 (5)	0.63807 (6)	0.0153 (2)	
Ho2	1.0000	0.0000	1.0000	0.0213 (4)	
I1	1.05135 (6)	−0.13025 (7)	0.83941 (7)	0.0190 (2)	
I2	1.31674 (7)	0.23705 (7)	0.50663 (8)	0.0242 (3)	
Fe1	1.0000	0.0000	0.5000	0.0132 (9)*	

### Atomic displacement parameters (Å^2^)

**Table:**

	*U*^11^	*U*^22^	*U*^33^	*U*^12^	*U*^13^	*U*^23^
Ho1	0.0155 (3)	0.0161 (3)	0.0147 (3)	0.0083 (3)	−0.0004 (2)	−0.0002 (2)
Ho2	0.0223 (5)	0.0223 (5)	0.0194 (7)	0.0111 (3)	0.000	0.000
I1	0.0202 (5)	0.0195 (5)	0.0183 (4)	0.0106 (4)	−0.0006 (3)	0.0010 (3)
I2	0.0167 (5)	0.0233 (5)	0.0260 (5)	0.0050 (4)	−0.0030 (3)	0.0060 (3)

### Geometric parameters (Å, °)

**Table:**

Ho1—Fe1	2.6056 (7)	Ho2—I1^vii^	3.0106 (9)
Ho1—I2	3.0722 (11)	Ho2—I1	3.0106 (9)
Ho1—I2^i^	3.1144 (11)	Ho2—I1^ii^	3.0106 (9)
Ho1—I1	3.1565 (11)	Ho2—I1^viii^	3.0106 (9)
Ho1—I1^ii^	3.1758 (11)	I1—Ho1^v^	3.1758 (11)
Ho1—I2^iii^	3.3116 (11)	I2—Ho1^iv^	3.1144 (11)
Ho1—Ho1^i^	3.6394 (11)	I2—Ho1^iii^	3.3116 (11)
Ho1—Ho1^iv^	3.6394 (11)	Fe1—Ho1^ix^	2.6056 (7)
Ho1—Ho1^v^	3.7297 (12)	Fe1—Ho1^iv^	2.6056 (7)
Ho1—Ho1^ii^	3.7297 (12)	Fe1—Ho1^ii^	2.6056 (7)
Ho2—I1^v^	3.0106 (9)	Fe1—Ho1^i^	2.6056 (7)
Ho2—I1^vi^	3.0106 (9)	Fe1—Ho1^v^	2.6056 (7)
			
Fe1—Ho1—I2	100.19 (3)	I2^iii^—Ho1—Ho1^ii^	133.68 (2)
Fe1—Ho1—I2^i^	99.12 (3)	Ho1^i^—Ho1—Ho1^ii^	90.0
I2—Ho1—I2^i^	89.813 (17)	Ho1^iv^—Ho1—Ho1^ii^	59.176 (12)
Fe1—Ho1—I1	98.41 (3)	Ho1^v^—Ho1—Ho1^ii^	60.0
I2—Ho1—I1	161.02 (3)	I1^v^—Ho2—I1^vi^	180.0
I2^i^—Ho1—I1	90.95 (3)	I1^v^—Ho2—I1^vii^	88.96 (2)
Fe1—Ho1—I1^ii^	97.93 (3)	I1^vi^—Ho2—I1^vii^	91.04 (2)
I2—Ho1—I1^ii^	88.28 (3)	I1^v^—Ho2—I1	91.04 (2)
I2^i^—Ho1—I1^ii^	162.91 (3)	I1^vi^—Ho2—I1	88.96 (2)
I1—Ho1—I1^ii^	85.44 (4)	I1^vii^—Ho2—I1	180.0
Fe1—Ho1—I2^iii^	177.02 (3)	I1^v^—Ho2—I1^ii^	91.04 (2)
I2—Ho1—I2^iii^	81.94 (3)	I1^vi^—Ho2—I1^ii^	88.96 (2)
I2^i^—Ho1—I2^iii^	82.92 (3)	I1^vii^—Ho2—I1^ii^	88.96 (2)
I1—Ho1—I2^iii^	79.33 (3)	I1—Ho2—I1^ii^	91.04 (2)
I1^ii^—Ho1—I2^iii^	80.00 (3)	I1^v^—Ho2—I1^viii^	88.96 (2)
Fe1—Ho1—Ho1^i^	45.702 (10)	I1^vi^—Ho2—I1^viii^	91.04 (2)
I2—Ho1—Ho1^i^	99.07 (3)	I1^vii^—Ho2—I1^viii^	91.04 (2)
I2^i^—Ho1—Ho1^i^	53.43 (2)	I1—Ho2—I1^viii^	88.96 (2)
I1—Ho1—Ho1^i^	96.59 (2)	I1^ii^—Ho2—I1^viii^	180.00 (3)
I1^ii^—Ho1—Ho1^i^	143.57 (2)	Ho2—I1—Ho1	91.20 (3)
I2^iii^—Ho1—Ho1^i^	136.24 (3)	Ho2—I1—Ho1^v^	90.83 (3)
Fe1—Ho1—Ho1^iv^	45.702 (10)	Ho1—I1—Ho1^v^	72.17 (3)
I2—Ho1—Ho1^iv^	54.50 (2)	Ho1—I2—Ho1^iv^	72.07 (3)
I2^i^—Ho1—Ho1^iv^	96.45 (3)	Ho1—I2—Ho1^iii^	98.06 (3)
I1—Ho1—Ho1^iv^	144.05 (2)	Ho1^iv^—I2—Ho1^iii^	170.08 (3)
I1^ii^—Ho1—Ho1^iv^	96.25 (2)	Ho1^ix^—Fe1—Ho1	180.00 (2)
I2^iii^—Ho1—Ho1^iv^	136.44 (2)	Ho1^ix^—Fe1—Ho1^iv^	91.403 (19)
Ho1^i^—Ho1—Ho1^iv^	61.65 (2)	Ho1—Fe1—Ho1^iv^	88.597 (19)
Fe1—Ho1—Ho1^v^	44.298 (10)	Ho1^ix^—Fe1—Ho1^ii^	88.597 (19)
I2—Ho1—Ho1^v^	144.47 (2)	Ho1—Fe1—Ho1^ii^	91.403 (19)
I2^i^—Ho1—Ho1^v^	96.42 (3)	Ho1^iv^—Fe1—Ho1^ii^	88.597 (19)
I1—Ho1—Ho1^v^	54.16 (2)	Ho1^ix^—Fe1—Ho1^i^	91.403 (19)
I1^ii^—Ho1—Ho1^v^	94.97 (2)	Ho1—Fe1—Ho1^i^	88.597 (19)
I2^iii^—Ho1—Ho1^v^	133.49 (2)	Ho1^iv^—Fe1—Ho1^i^	91.403 (19)
Ho1^i^—Ho1—Ho1^v^	59.176 (12)	Ho1^ii^—Fe1—Ho1^i^	180.0
Ho1^iv^—Ho1—Ho1^v^	90.0	Ho1^ix^—Fe1—Ho1^v^	88.597 (19)
Fe1—Ho1—Ho1^ii^	44.298 (10)	Ho1—Fe1—Ho1^v^	91.403 (19)
I2—Ho1—Ho1^ii^	95.36 (3)	Ho1^iv^—Fe1—Ho1^v^	180.00 (3)
I2^i^—Ho1—Ho1^ii^	143.40 (2)	Ho1^ii^—Fe1—Ho1^v^	91.404 (19)
I1—Ho1—Ho1^ii^	95.30 (2)	Ho1^i^—Fe1—Ho1^v^	88.597 (19)
I1^ii^—Ho1—Ho1^ii^	53.68 (2)		

Symmetry codes: (i) *y*+1, −*x*+*y*+1, −*z*+1; (ii) −*y*+1, *x*−*y*−1, *z*; (iii) −*x*+8/3, −*y*+1/3, −*z*+4/3; (iv) *x*−*y*, *x*−1, −*z*+1; (v) −*x*+*y*+2, −*x*+1, *z*; (vi) *x*−*y*, *x*−1, −*z*+2; (vii) −*x*+2, −*y*, −*z*+2; (viii) *y*+1, −*x*+*y*+1, −*z*+2; (ix) −*x*+2, −*y*, −*z*+1.
